# Supplementary material for: People with heart failure, sarcopenia and chagas disease: A systematic review and meta-analysis
Source: PLoS Negl Trop Dis. 2025 Nov 10;19(11):e0013699. doi: 10.1371/journal.pntd.0013699 (PMC12599930; doi:10.1371/journal.pntd.0013699)
Supplement: S1 Text — (DOCX) [file pntd.0013699.s001.docx]

**Supplementary Material Text**

*Search strategy*

Supplementary Table A. Search strategy

| Database | Search strategy |
| --- | --- |
| EMBASE | **'heart failure'**/exp OR **'heart failure'** OR **'systolic dysfunction'**/exp OR **'systolic dysfunction'** OR **'diastolic dysfunction'**/exp OR **'diastolic dysfunction'** OR **'congestive heart failure'**/exp OR **'congestive heart failure'** OR **'diastolic heart failure'**/exp OR **'diastolic heart failure'** OR **'heart failure'**:ab,ti OR**'heart congestion'**:ab,ti OR **'systolic dysfunction'**:ab,ti OR **'systolic failure'**:ab,ti OR **'diastolic dysfunction'**:ab,ti  OR **'diastolic failure'**:ab,ti OR **'ejection fraction'**:ab,ti  OR **'chagas disease'**:ab,ti OR **chagas**:ab,ti OR **'tripanosoma cruzi'**:ab,ti **'chagas disease'**/exp OR **'chagas disease'**OR **'chagas cardiomyopathy'**/exp OR **'chagas cardiomyopathy'**    AND    **'sarcopenia'**/exp OR **'sarcopenia'**  OR  **sarcopenia**:ab,ti |
| PUBMED | **#1**  (heart failure[Title/Abstract]) OR (cardiac failure[Title/Abstract]) OR (heart disease[Title/Abstract]) OR (systolic failure[Title/Abstract]) OR (diastolic failure[Title/Abstract]) OR (ejection fraction[Title/Abstract]) OR "Heart Failure"[Mesh] OR "Heart Failure, Diastolic"[Mesh] OR "Heart Failure, Systolic"[Mesh] OR (Chagas[Title/Abstract]) OR (Trypanosomiasis[Title/Abstract]) OR (Trypanosoma cruzi[Title/Abstract]) OR ( "Chagas Disease"[Mesh] OR "Chagas Cardiomyopathy"[Mesh] )    AND  **#2**  ("Sarcopenia"[Mesh]) OR (sarcopenia [Title/Abstract]) |
| BIREME | (heart failure) OR (cardiac failure) OR (heart disease) OR (systolic failure) OR (diastolic failure) OR (ejection fraction) OR (Chagas) OR (mh:(Heart Failure)) OR (mh:(Heart Failure, Diastolic)) OR (mh:(Heart Failure, Systolic)) OR (Trypanosomiasis) OR (Trypanosoma cruzi) OR (mh:(Chagas Disease)) OR (mh:(Chagas Cardiomyopathy))  AND  (Sarcopenia) OR (mh:(Sarcopenia)) |

*Data extracted*

Table B. Data extracted

| Data extracted |
| --- |
| the name of the authors; year of publication; study design; country of the study; study settings; number of participants in each group [PLHF and controls(when present)]; arm losses; age (mean and standard deviation); body mass index (BMI, mean and standard deviation); gender; ejection fraction (%); New York Heart Association heart failure class (NYHA); number of participants with Chagas Disease; muscle mass assessment method; kind of muscle mass assessment (total lean mass, appendicular lean mass; free fat mass); method of strength evaluation; sarcopenia frequency; and criterion used for the sarcopenia diagnosis. |

The above data were extracted from each article using an Excel spreadsheet:

*Study quality*

Table C. Evaluation of the quality of the included studies according to the New Castle Ottawa scale.

| **Author** | **Year** | **Selection** | **Comparability** | **Outcome** | **Total** |
| --- | --- | --- | --- | --- | --- |
| Formiga et al.[1] | 2024 | **** | * | *** | 8 |
| Bieger et al.[2] | 2023 | **** |  | *** | 7 |
| Valdiviesso et al.[3] | 2022 | **** | ** | *** | 9 |
| Karim et al.[4] | 2022 | *** | ** | *** | 8 |
| Cvjetan et al.[5] | 2022 | *** | ** | ** | 7 |
| Karim et al. [6] | 2022 | *** | ** | *** | 8 |
| Fonseca et al.[7] | 2022 | *** | ** | *** | 8 |
| Pinijmung et al[8] | 2022 | ** | ** | *** | 7 |
| Fonseca et al.[9] | 2020 | *** | ** | *** | 8 |
| Fonseca et al.[10] | 2020 | *** | ** | *** | 8 |
| Telfer et al.[11] | 2020 | ** | ** | *** | 7 |
| Sun et al.[12] | 2020 | ** | ** | *** | 7 |
| Canteri et al.[13] | 2020 | *** | ** | *** | 8 |
| Loncar et al.[14] | 2019 | *** | ** | ** | 7 |
| Nozaki et al.[15] | 2019 | *** | ** | ** | 7 |
| Fonseca et al.[16] | 2019 | *** | ** | *** | 8 |
| Gulyaev et al.[17] | 2019 | ** | ** | *** | 7 |
| Emami et al.[18] | 2019 | *** | ** | *** | 8 |
| Dos Santos et al.[19] | 2019 | *** | ** | *** | 9 |
| Watanabe et al.[20] | 2019 |  |  |  | 0 |
| Hajahmadi et al.[21] | 2017 | ** | ** | *** | 7 |
| Molinero-Abad et al.[22] | 2017 | **** |  | ** | 6 |
| Obata et al. [23] | 2015 | ** | ** | *** | 7 |
| Haykowsky et al.[24] | 2015 | *** | ** | *** | 8 |
| Landi et al.[25] | 2013 | **** | ** | *** | 9 |

Figure A. Forest plot of the frequency of Sarcopenia in people living with heart failure by Sarcopenia Definition


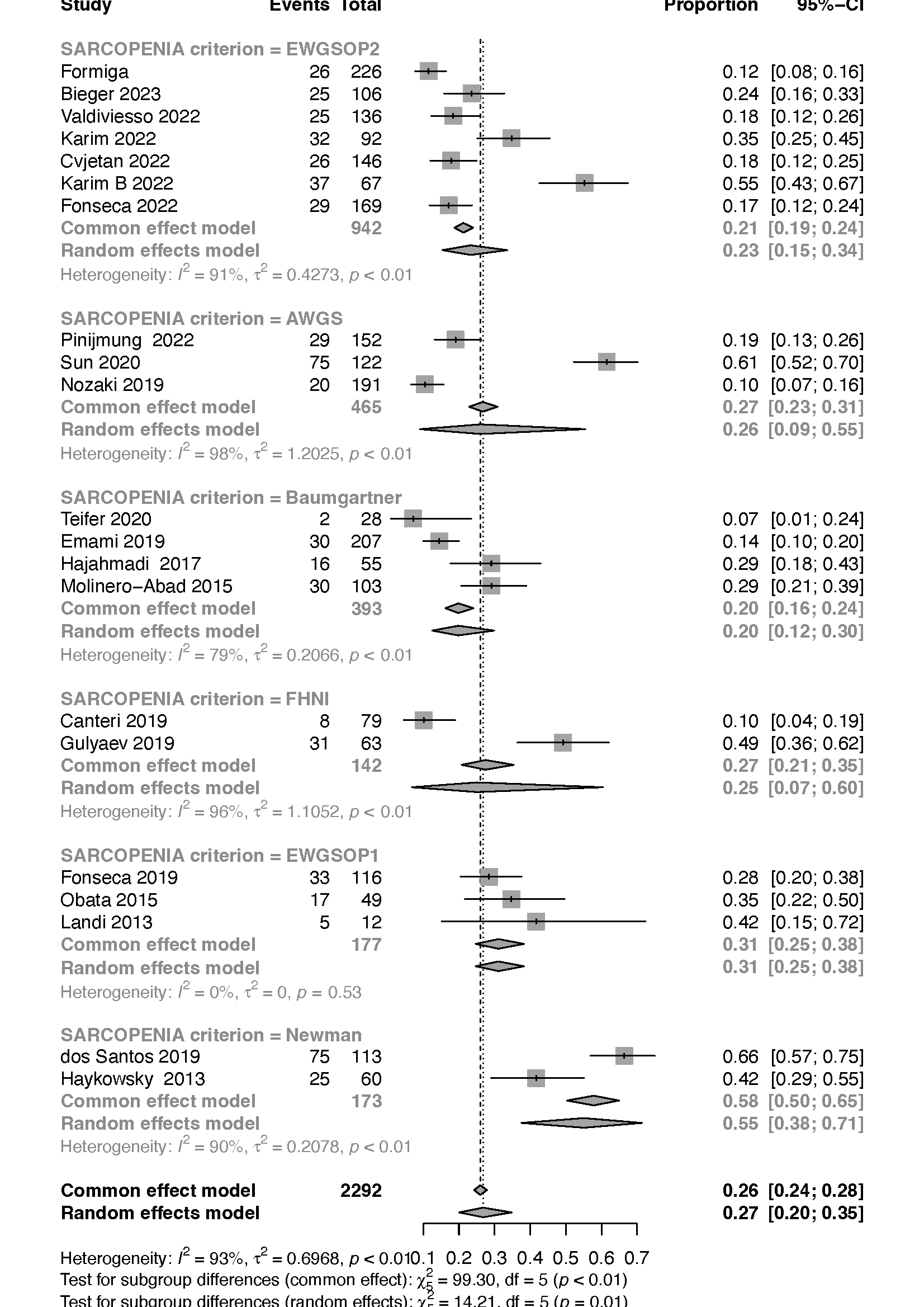


Footnote:

EWGSOP2 = sarcopenia definition proposed by European Working Group on Sarcopenia in Older People in 2018

AWGS = sarcopenia definition proposed by Asian Working Group for Sarcopenia in 2014

Baumgartner [26] = sarcopenia definition proposed by Baumgartner et al. in 1998

EWGSOP1 = = sarcopenia definition proposed by European Working Group on Sarcopenia in Older People in 2010

FHNI = sarcopenia definition proposed by Foundation for the National Institutes of Health in 2014

Newman [27]= sarcopenia definition proposed by Newman et al. in 2003

Table D. Live-one-out analysis on the frequency of Sarcopenia metanalysis

|  | **Pooled frequency of sarcopenia (95%-CI)** | **I², %** |
| --- | --- | --- |
| All | 25.68 (19.02; 33.70) | 92.0% |
| Omitting Formiga | 26.50 (24.42; 28.70) | 90.8% |
| Omitting Bieger 2023 | 24.74 (22.78; 26.82) | 92.0% |
| Omitting Valdiviesso 2022 | 25.17 (23.18; 27.28) | 91.8% |
| Omitting Karim 2022 | 24.15 (22.21; 26.20) | 91.9% |
| Omitting Cvjetan 2022 | 25.26 (23.26; 27.38) | 91.8% |
| Omitting Karim (B) 2022 | 23.53 (21.62; 25.55) | 90.9% |
| Omitting Fonseca 2022 | 25.43 (23.41; 27.56) | 91.8% |
| Omitting Pinijmung 2022 | 25.18 (23.17; 27.29) | 91.8% |
| Omitting Teifer 2020 | 24.95 (23.01; 26.98) | 91.8% |
| Omitting Sun 2020 | 22.09 (20.20; 24.10) | 87.9% |
| Omitting Canteri 2019 | 25.32 (23.36; 27.40) | 91.6% |
| Omitting Nozaki 2019 | 26.31 (24.25; 28.48) | 90.9% |
| Omitting Gulyaev 2019 | 23.81 (21.90; 25.84) | 91.4% |
| Omitting Hajahmadi 2017 | 24.54 (22.61; 26.58) | 92.0% |
| Omitting Molinero-Abad 2015 | 24.42 (22.46; 26.44) | 92.0% |
| Omitting Obata 2015 | 24.41 (22.48; 26.44) | 92.0% |
| Omitting Haykowsky 2013 | 24.11 (22.19; 26.58) | 91.8% |
| Omitting Landi 2013 | 24.57 (22.65; 26.58) | 92.0% |

Figure B. Funnel Plot on the frequency of sarcopenia in people living with heart failure metanalysis


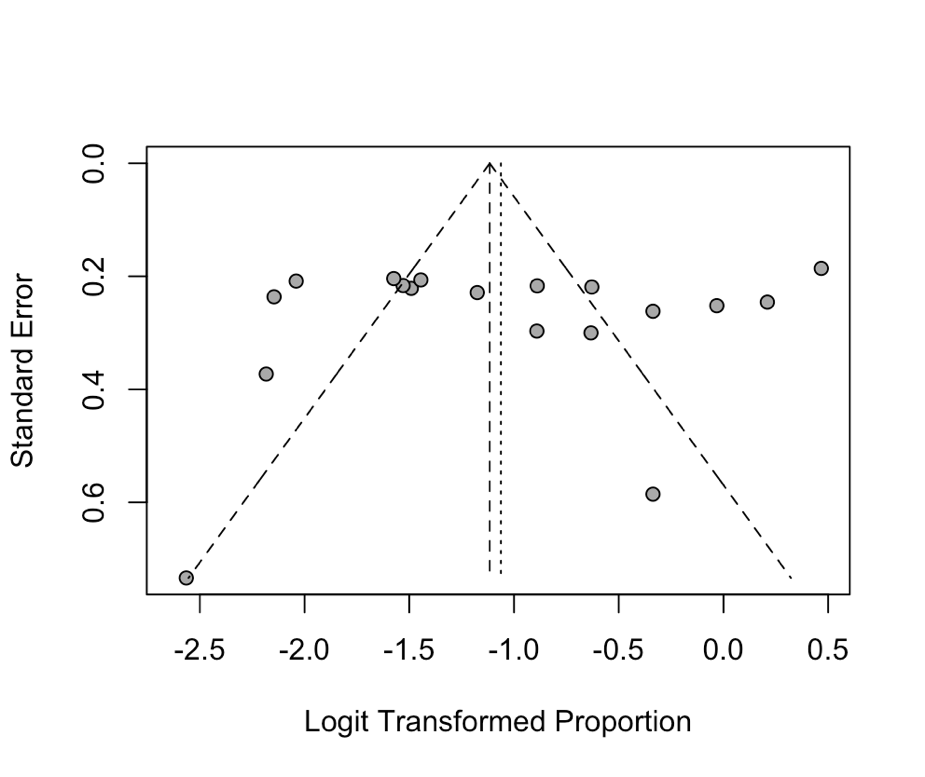


Figure C. Funnel Plot on the OR of sarcopenia in people living with heart failure metanalysis

Table E. Live-one-out analysis on the OR of sarcopenia in people living with heart failure metanalysis

|  | **Pooled OR (95%-CI)** | **I^2** |
| --- | --- | --- |
| All | 2.3 (1.14; 4.81) | 77.2% |
| Omitting Formiga 2024 | 3.7 (2.22; 6.11) | 0.0% |
| Omitting Karim 2022 | 1.8 (0.83; 3.80) | 53.6% |
| Omitting Canteri 2019 | 2.2 (0.90; 5.43) | 83.8% |
| Omitting Landi 2013 | 2.3 (0.93; 5.64) | 84.2% |

Table F. GRADE classification for the outcomes

| Outcome | Number of studies |  |  | | | | | | | GRADE* |
| --- | --- | --- | --- | --- | --- | --- | --- | --- | --- | --- |
|  |  | Risk of bias | Inconsistency | Indirectness | Imprecision | Publication bias | Large effect | Dose–effect response | Residual confounding  with antagonizing  effect |  |
| Prevalence of Sarcopenia in HF^a^ | 21 | Low | Yes | No | No | No | Yes  26.77 % (95%CI 20.06, 34.76) | No | No | ⨁⨁⨁◯ |
| OR of Sarcopenia in HF^a^ | 4 | Low | Yes | No | No | No | No | No | No | ⨁⨁◯◯ |
|  |  |  |  |  |  |  |  |  |  |  |
| Prevalence of Sarcopenia in PLHFC | 2 | Low | No | No | No | Unknown | No | No | No | ⨁⨁◯◯ |
| OR of Sarcopenia in PLHFC | 2 | Low | Yes | No | no | Unknown | No | No | No | ⨁◯◯◯ |
|  |  |  |  |  |  |  |  |  |  |  |

*In this systematic review and metanalysis the GRADE starts at ⨁⨁⨁◯ due to the observational design of the included studies

^a^HF = heart failure

^b^PLHFC = people living with heart failure due to Chagas Disease

**References included in the systematic review**

1. Formiga F, Moreno-Gonzalez R, Corsonello A, Mattace-Raso F, Carlsson AC, Arnlov J, et al. Prevalence of Sarcopenia in Chronic Heart Failure and Modulating Role of Chronic Kidney Disease. Gerontology. 2024;70(5):507-16. Epub 2024/02/07. doi: 10.1159/000536465. PubMed PMID: 38320538.

2. Bieger P, Sangali TD, Ribeiro ECT, Schweigert Perry ID, Souza GC. Association of phase angle values and sarcopenia in older patients with heart failure. Nutr Clin Pract. 2023;38(3):672-85. Epub 2023/02/24. doi: 10.1002/ncp.10956. PubMed PMID: 36815519.

3. Valdiviesso R, Sousa-Santos AR, Azevedo LF, Moreira E, Amaral TF, Silva-Cardoso J, et al. Statins are associated with reduced likelihood of sarcopenia in a sample of heart failure outpatients: a cross-sectional study. BMC Cardiovasc Disord. 2022;22(1):356. Epub 2022/08/06. doi: 10.1186/s12872-022-02804-5. PubMed PMID: 35931947; PubMed Central PMCID: PMCPMC9354359.

4. Karim A, Muhammad T, Shah I, Khan J, Qaisar R. A multistrain probiotic reduces sarcopenia by modulating Wnt signaling biomarkers in patients with chronic heart failure. J Cardiol. 2022;80(5):449-55. Epub 2022/06/25. doi: 10.1016/j.jjcc.2022.06.006. PubMed PMID: 35750555.

5. Cvjetan RE, J; Gojkovic, F; Aleksic, J. SARCOPENIA IN PATIENTS WITH HEART FAILURE in: World Congress on Osteoporosis, Osteoarthritis and Musculoskeletal Diseases (WCO-IOF-ESCEO 2022). Aging Clin Exp Res. 2022;34(Suppl 1):111. Epub 2022/08/26. doi: 10.1007/s40520-022-02147-3. PubMed PMID: 36008648; PubMed Central PMCID: PMCPMC9411039.

6. Karim A, Muhammad T, Shah I, Khan J, Qaisar R. Relationship of Haptoglobin Phenotypes With Sarcopaenia in Patients With Congestive Heart Failure. Heart Lung Circ. 2022;31(6):822-31. Epub 2022/02/20. doi: 10.1016/j.hlc.2022.01.003. PubMed PMID: 35181229.

7. Fonseca GWP, Bispo HN, Rondon E, Dos Santos MR, De Souza FR, Da Costa MJA, et al. Association of sarcopenia and oxygen uptake efficiency slope in male patients with heart failure: The skeletal muscle hypothesis in practice. European Journal of Preventive Cardiology. 2022;29(Supplement_1):zwac056.11. doi: 10.1093/eurjpc/zwac056.011.

8. Pinijmung PY, K; * Buakhamsri, A. Prevalence and Impact of Sarcopenia in Heart Failure: A Cross-Sectional Study. The Open Cardiovascular Medicine Journal. 2022;16:e187419242202240.

9. Fonseca G, Garfias Macedo T, Ebner N, Dos Santos MR, de Souza FR, Mady C, et al. Muscle mass, muscle strength, and functional capacity in patients with heart failure of Chagas disease and other aetiologies. ESC Heart Fail. 2020;7(5):3086-94. Epub 2020/08/30. doi: 10.1002/ehf2.12936. PubMed PMID: 32860353; PubMed Central PMCID: PMCPMC7524247.

10. Fonseca G, Dos Santos MR, de Souza FR, Takayama L, Rodrigues Pereira RM, Negrao CE, et al. Discriminating sarcopenia in overweight/obese male patients with heart failure: the influence of body mass index. ESC Heart Fail. 2020;7(1):84-91. Epub 2019/12/27. doi: 10.1002/ehf2.12545. PubMed PMID: 31877587; PubMed Central PMCID: PMCPMC7083394.

11. Telfer L, Chery J, Lawrence M, Haynes A, Vest AR. Evaluation of Skeletal Muscle Ultrasound For The Assessment of Body Composition In Healthy Controls And Patients With Heart Failure. Journal of Cardiac Failure. 2020;26(10, Supplement):S42. doi: <https://doi.org/10.1016/j.cardfail.2020.09.126>.

12. Sun YC, Y; Ma, Q. Elderly Chronic Heart Failure Patients with Sarcopenia had

Lower MNA-SF Scores and Lower Serum Albumin Level. Journal of the American Geriatrics Society. 2020;68(S1):S50. doi: <https://doi.org/10.1111/jgs.16431>.

13. Canteri AL, Gusmon LB, Zanini AC, Nagano FE, Rabito EI, Petterle RR, et al. Sarcopenia in heart failure with reduced ejection fraction. Am J Cardiovasc Dis. 2019;9(6):116-26. Epub 2020/01/24. PubMed PMID: 31970027; PubMed Central PMCID: PMCPMC6971421.

14. Loncar G, Bozic B, Von Haehling S, Cvetinovic N, Lainscak M, Dungen HD, et al. P4541Sarcopenia in non-cachectic males with heart failure. European Heart Journal. 2019;40(Supplement_1):ehz745.0932. doi: 10.1093/eurheartj/ehz745.0932.

15. Nozaki Y, Yamaji, M., Nishiguchi, S., Fukutani, N., Tashiro, Y., Shirooka, H., Hirata, H., Yamaguchi, M., Tasaka, S., Matsubara, K., Matsushita, T., Hikita, Y., Oya, K., Aoyama, T., & Mabuchi, H. . Sarcopenia predicts adverse outcomes in an elderly outpatient population with New York heart association class II–IV heart failure: A prospective cohort study. . Aging Medicine and Healthcare. 2019;10(2):53-61.

16. Fonseca G, Santos MRD, Souza FR, Costa M, Haehling SV, Takayama L, et al. Sympatho-Vagal Imbalance is Associated with Sarcopenia in Male Patients with Heart Failure. Arq Bras Cardiol. 2019;112(6):739-46. Epub 2019/04/11. doi: 10.5935/abc.20190061. PubMed PMID: 30970141; PubMed Central PMCID: PMCPMC6636362 article was reported.

17. Gulyaev NI, Akhmetshin IM, Gordienco AV, Kulikov AN. Possibilities of Ultrasound Diagnosis of Sarcopenia in Elderly Patients with Chronic Heart Failure. Advances in Gerontology. 2020;10(2):182-6. doi: 10.1134/S207905702002006X.

18. Emami A, Saitoh M, Valentova M, Sandek A, Evertz R, Ebner N, et al. Comparison of sarcopenia and cachexia in men with chronic heart failure: results from the Studies Investigating Co-morbidities Aggravating Heart Failure (SICA-HF). Eur J Heart Fail. 2018;20(11):1580-7. Epub 2018/08/31. doi: 10.1002/ejhf.1304. PubMed PMID: 30160804.

19. Dos Santos MR, Fonseca GWP, Sherveninas L, Souza FR, Battaglia Filho AC, Pereira RMR, et al. P4539Android to gynoid fat ratio and its association with functional capacity in male patients with heart failure. European Heart Journal. 2019;40(Supplement_1):ehz745.0930. doi: 10.1093/eurheartj/ehz745.0930.

20. Watanabe T, Narumi T, Kubota I. SY4-3 - Impact of Sarcopenia on the Prognosis in Heart Failure. Journal of Cardiac Failure. 2017;23(10, Supplement):S8. doi: <https://doi.org/10.1016/j.cardfail.2017.08.018>.

21. Hajahmadi M, Shemshadi S, Khalilipur E, Amin A, Taghavi S, Maleki M, et al. Muscle wasting in young patients with dilated cardiomyopathy. J Cachexia Sarcopenia Muscle. 2017;8(4):542-8. Epub 2017/03/03. doi: 10.1002/jcsm.12193. PubMed PMID: 28251827; PubMed Central PMCID: PMCPMC5566643.

22. Molinero-Abad S, Soto-Célix M, Riego-Valledor A, Blanco-de-Morentín A, Marti-Bonmati E, Mijan-de-la-Torre A. SUN-PP145: Diagnosis of Sarcopenia is Associated with a Lower Survival in Chronic Heart Failure Patients (CHF). Clinical Nutrition. 2015;34:S77. doi: <https://doi.org/10.1016/S0261-5614(15)30295-8>.

23. Obata HI, T; Watanabe, W; Mitsuma, W; Tomii, T; Sakai, T; Uehara, A Minamino, T. Characteristics of sarcopenia in patients with chronic heart failure. European Journal of Preventive Cardiology. 2015;22 (1_suppl):S51.

24. Haykowsky MJ, Brubaker PH, Morgan TM, Kritchevsky S, Eggebeen J, Kitzman DW. Impaired aerobic capacity and physical functional performance in older heart failure patients with preserved ejection fraction: role of lean body mass. J Gerontol A Biol Sci Med Sci. 2013;68(8):968-75. Epub 2013/03/26. doi: 10.1093/gerona/glt011. PubMed PMID: 23525477; PubMed Central PMCID: PMCPMC3712362.

25. Landi F, Cruz-Jentoft AJ, Liperoti R, Russo A, Giovannini S, Tosato M, et al. Sarcopenia and mortality risk in frail older persons aged 80 years and older: results from ilSIRENTE study. Age Ageing. 2013;42(2):203-9. Epub 2013/01/17. doi: 10.1093/ageing/afs194. PubMed PMID: 23321202.

26. Baumgartner RN, Koehler KM, Gallagher D, Romero L, Heymsfield SB, Ross RR, et al. Epidemiology of sarcopenia among the elderly in New Mexico. Am J Epidemiol. 1998;147(8):755-63. Epub 1998/04/29. doi: 10.1093/oxfordjournals.aje.a009520. PubMed PMID: 9554417.

27. Newman AB, Kupelian V, Visser M, Simonsick E, Goodpaster B, Nevitt M, et al. Sarcopenia: alternative definitions and associations with lower extremity function. J Am Geriatr Soc. 2003;51(11):1602-9. Epub 2003/12/23. doi: 10.1046/j.1532-5415.2003.51534.x. PubMed PMID: 14687390.

**LIST OF LEGENDS**

HF = heart failure

HF-C = heart failure due to Chagas disease

HF-NC = heart failure from other causes

PRISMA = Preferred Reporting Items for Systematic Reviews and Meta-analyses

PROSPERO = The review protocol was registered at the University of York database

MEDLINE = Medical Literature Analysis and Retrieval System Online

EMBASE = the Excerpta Medica dataBASE

BIREME = Regional Library of Medicine

NOS = Newcastle-Ottawa scale

EWGSOP = European Working Group on Sarcopenia in Older People

AWGS = Asian Working Group for Sarcopenia

FNIH = Foundation for the National Institutes of Health
