# Supplementary material for: People with heart failure, sarcopenia and chagas disease: A systematic review and meta-analysis
Source: PLoS Negl Trop Dis. 2025 Nov 10;19(11):e0013699. doi: 10.1371/journal.pntd.0013699 (PMC12599930; doi:10.1371/journal.pntd.0013699)
Supplement: Attachment — Submitted filename: Response-PLOS Neglected disease.docx [file pntd.0013699.s004.docx]

28^th^ August 2025

Dear Andrés F. Henao-Martínez, M.D.
Academic Editor
PLOS Neglected Tropical Diseases

We would like to thank the editor and reviewers for their thoughtful comments on this paper. Please find enclosed a new version of the paper entitled **“PEOPLE WITH HEART FAILURE, SARCOPENIA AND CHAGAS DISEASE: A SYSTEMATIC REVIEW AND META-ANALYSIS”** (manuscript ID PNTD-D-25-00641), revised according to the comments received.

We hope this revised version will now be suitable for publication in your journal. Please find below our point-by-point response to the comments.

Yours sincerely.

Melissa Premaor

**RESPONSE TO REVIEWERS**

**Methods**

*Reviewer #1:*

1) *In the study design, mention that it is also a meta-analysis.*

Thank you for the comment. We included that in the methods.

2) *Describe acronym meaning PRISMA- Preferred Reporting Items for Systematic Reviews and Metanalyses (PRISMA).*

Thank you for the comment. We included the acronym's meaning in the methods.

It now reads: “This study was designed as a systematic review and meta-analysis. It has followed the PRISMA- Preferred Reporting Items for Systematic Reviews and Metanalyses (PRISMA) Guidelines.”

3) *If the aim is to assess sarcopenia and HF, it is not understandable to exclude hospitalized patients.*

We thank the reviewer for the comment. We decided to exclude inpatients due to the chance that they might have some degree of acute sarcopenia. The concept of acute sarcopenia is increasingly accepted, and it is likely caused by acute immobilization and worsening of inflammation that occurs in inpatients. The EWGSOP2 itself defines this concept and recommends that acute sarcopenia be considered a separate disorder (CRUZ-JENTOFT; BAHAT; BAUER; BOIRIE *et al.*, 2019). Supporting this hypothesis, the systematic review and meta-analysis by Zhang et al. found a much higher prevalence of sarcopenia in inpatients compared to outpatients [55.0% (95%CI 43.0%, 66.0%) vs. 26.0% (95%CI 16.0%, 37.0%)](ZHANG; ZHANG; NI; YUAN *et al.*, 2021).

4) *“The previously defined terms for each database are described in the supplementary material” . number the material in the text for easy identification, and keep it consistent with the citation in the text*

Thank you for the comment. We have numbered the material and revised the text citation for consistency.

Reviewer #2:

1) *The objective and hypothesis are well-described. A systematic review is appropriate to summarize* *evidence. The selected papers met the pre-established inclusion/exclusion criteria based on the clinical definition. However, despite the 25 reviewed articles included >2500 heart failure patients, the subgroup of Chagas disease (CD) patients is underpowered (only 2-3 studies assessed sarcopenia in CD patients), limiting conclusive results for the proposed comparison. No concern about ethical or regulatory requirements.*

We thank the reviewer for the comment. We agree that there are too few studies evaluating Sarcopenia in heart failure due to Chagas disease and that our study is hypothesis-generating. Nevertheless, our study is important in highlighting the significance of this problem and outlining the existing knowledge gap.

*Reviewer #3:*

1) *The study was conducted in accordance with PRISMA guidelines, and its protocol was registered in PROSPERO, reflecting a commitment to transparency and methodological rigor. Key aspects of the analysis—such as eligibility criteria, data sources, and statistical methods—are aligned with best practices for systematic reviews and meta-analyses. The authors applied appropriate inclusion and exclusion criteria, focusing on adult patients with heart failure (HF) of any etiology, including Chagas disease, while explicitly excluding studies involving specific subgroups, thereby reducing the risk of selection bias. The methods section demonstrates a structured and comprehensive approach to study selection, data extraction, and quality assessment.*

We thank the reviewer for the comment.

*Reviewer #4: (No Response)*

**Results**

Reviewer #1:

1) *Studies that evaluated the same population should be excluded as they may overestimate the data, including prevalence. Perhaps the ideal would be to include only the additional information from the cohort, and not the prevalence. How did you manage to avoid this bias?*

*Cvjetan et al.[14] is an update from the conference paper by Loncar et al.[15*

*Fonseca et al.[16], Fonseca et al.[17, 18], Emmani et al.[19], and dos Santos et al.[20] included subjects from the same cohort.*

We thank the reviewer for their comment. We adjusted the analysis in the results to include only the study with the largest number of participants in the meta-analysis. We updated Figure 2 and all the results related to this analysis.

We kept the studies in Table 1 because they provide different information. The figure comparing the different sarcopenia criteria was moved to the supplementary material.

The results now read: “The pooled analysis for sarcopenia included 21 studies. The prevalence of sarcopenia in HF patients was 25.68 % (95%CI 19.02, 33.70), Figure 2; ⨁⨁⨁◯. However, the frequency of Sarcopenia assessed by the different criteria has shown a significant difference, p-value = 0.01, Supplementary Figure 1. The lowest prevalence was evaluated by the Baumgartner operational definition [19.79% (95%CI 12.49, 29.91), Supplementary Figure 1.] while the highest was the frequency evaluated by the Newman operational definition [55.01% (95%CI 37.55, 71.31), Supplementary Figure 1]. The prevalence of Sarcopenia evaluated by the EWGSOP2 definition was 23.27% (95%CI 15.39, 33.58), Supplementary Figure 1.

We carried out the live-one-out analysis (Supplementary Table 4); nonetheless, the pooled results were not affected by this procedure, and no single article could explain the source of the heterogeneity. Further, the p-value for the publication bias evaluated by the Begg test was 0.722. The funnel plot is displayed in Supplementary Figure 2. Even though it shows symmetry, most studies have a large effect size.”

2) *In table 2 there is a blank row*

We thank the reviewer for the comment. It was excluded.

3) *"One study has estimated muscle mass using the Forbes formula”. reference the study. Was it excluded from the data analysis or review? If it was excluded from the review, it would be interesting to describe it in the flow of included studies*

The study that estimated muscle mass using the Forbes formula was conducted by Watanabe et al.(WATANABE; NARUMI; KUBOTA, 2017). The reference was added to the text.

The study was included in the systematic review but not in the meta-analysis. This information was clarified in the text.

It now reads: “One study has estimated muscle mass using the Forbes formula (Table 2) [21]. As the Forbes formula estimates muscle mass through the serum levels of creatinine and there is some controversy in this method of estimation, this latter study was not included in the meta-analysis.”

*Reviewer #2:*

1) *Multiple diagnostic criteria for sarcopenia were included, which are not directly comparable. Despite of this, the statistical analysis was correct, including heterogeneity assessment. The pooled OR for CH patients with sarcopenia was not statistically significant, with a wide confidence interval, indicating high uncertainty.*

We agree that the different criteria for Sarcopenia are not directly comparable. Our intention was to confirm the different prevalence values for the various criteria. Once we confirmed it, we kept it in the results to highlight these differences for awareness. Sarcopenia is a new field of study, and the use of these definitions is not yet fully established.

2) *The exclusion of hospitalized patients, aimed to reduce confounding from acute illness, also limits the generalizability.*

We agree with the reviewer that our results can only be generalized for outpatients with heart failure. However, we consider this selection of patients important because we believe that measures to prevent Sarcopenia in this population can be studied. If proven effective, outpatient care can be expanded.

*Reviewer #3:*

1) *The results presented are fully consistent with the analysis plan described in the methods section. All proposed procedures were executed. The presentation of the results is clear, structured, and comprehensive. The description details the selection processes, characteristics of the included studies, methods for assessing muscle mass and sarcopenia, and the comparison of sarcopenia prevalence between patients with and without heart failure, as well as between patients with Chagas disease-related heart failure and other etiologies.*

We thank the reviewer for the comment

*Reviewer #4:*

1) *I believe the authors should consider moving some of the topics currently presented in the supplementary material into the main body of the manuscript, in order to facilitate readers’ analysis and comprehension.*

We thank the reviewer for the suggestion. However, we decided not to move the results contained in the supplementary material to the body of the article due to its size. We standardized the presentation of the results and placed all the data from the supplementary material in tables or figures to facilitate understanding.

**Conclusions**

*Reviewer #1:*

1) *The confidence intervals for the odds ratio are similar between IC and Chagas-related IC. So I believe they can be considered similar, rather than higher. Moreover, the prevalence was slightly higher and was assessed in only two studies.*

We agree with the reviewer that there are very few studies. We believe the importance of our study lies in raising the hypothesis that Sarcopenia may be a problem in patients with heart failure due to Chagas disease and highlighting the need to study this topic in this population.

*Reviewer #2:*

1) *The conclusion that the risk of sarcopenia in HF Chagas patients might be higher than HF from other causes is not supported by the data, given the small number of patients and the wide OR. A more cautious statement should mention that the results sh*ould be a hypothesis-generating observation, not a finding.

We agree and have rewritten the text as suggested for better understanding.

The abstract conclusion now reads: “**Conclusions**: Patients with HF have an increased risk of developing sarcopenia. However, the prevalence of sarcopenia varies according to the definition used. Furthermore, c.”

The manuscript conclusion now reads: “In conclusion, our systematic review indicates that people with HF are at increased risk for sarcopenia. Further, we found that people with HF are 2.35 times more likely to present Sarcopenia when compared to people without HF. These findings have potential implications for the quality of life of patients with HF and should be considered in the care provided to these individuals. Our findings highlight the hypothesis that sarcopenia could be a problem in HF-C. Nevertheless, more studies are needed to evaluate sarcopenia in HF-C.”

2) *Also, it is important to acknowledge the geographical limitations of the data. The authors acknowledged that co-morbidities, frailty, chronic disease, etc, are relevant confounders and may overlap with sarcopenia, but this was not addressed in the data.*

We agree with the reviewer that these factors need to be assessed. However, the designs of the included studies did not allow us to assess them in this systematic review and meta-analysis. We believe that more studies with specific designs to assess these confounders are needed.

3) *The inconclusive results limit its public health relevance at this point.*

We agree with the reviewer, but we understand that more studies are needed to evaluate this problem, and therefore, more investment is also needed.

.

*Reviewer #3:*

1) *This systematic review presents consistent evidence that patients with heart failure—including those with Chagas disease—are at increased risk of developing sarcopenia, particularly when assessed using recent and more stringent diagnostic criteria. While the findings are limited by heterogeneity among studies and the relatively small number of investigations focused specifically on Chagas disease, this review provides important contributions to the current understanding of sarcopenia in neglected populations and identifies clear priorities for future research.*

We thank the reviewer for the comment.

*Reviewer #4:*

1) *The manuscript is well-structured and clearly written. Although the number of referenced studies is limited, the work remains methodologically sound and scientifically relevant. The authors are encouraged to continue advancing research in this field, particularly given the importance of understanding sarcopenia in the context of heart failure and neglected diseases such as Chagas disease.*

We thank the reviewer for the comment.

**Editorial and Data Presentation Modifications?**

*Reviewer #1:*

1) *“Among those infected, approximately 30% develop chronic cardiomyopathy with arrhythmias and heart failure”. • There is a recent systematic review that reports this percentage as 42%*

We thank the reviewer for the comment. The data was updated.

*Reviewer #2:*

1) *Clarify and temper the conclusions, which are just speculative.*

We thank the reviewer for the comment. It was done.

2) *Attempt to stratify results by diagnostic criteria and sarcopenia definitions. A supplementary table could present sarcopenia criteria in each study to facilitate readers.*

We thank the reviewer for the comment. These definitions are displayed in Table 2. Please find it bellow.

**Table 2.** Caption: Description of the methods used to evaluate the studies outcomes

| Author | Year | **Sarcopenia (number)** | **Sarcopenia criterion** | **Lean mass criterion** | **Lean mass techinique** | **Stength criteeion** | **Strengh techinique** | **Chagas Sarcopenia (number )** | **Comorbidities** |
| --- | --- | --- | --- | --- | --- | --- | --- | --- | --- |
| Formiga et al. | 2023 | 26 | EWGOSP 2 | ASMI | Bioimpedance | EWGOSP 2 | Handgrip | NI |  |
| Bieger et al. | 2023 | 25 | EWGOSP 2 | ASMI | Bioimpedance | EWGOSP 2 | Handgrip Five-Times-Sit-to-Stand Test | NI | HBP 69.4% DM 36.8% |
| Valdiviesso et al. | 2022 | 25 | EWGOSP 2 | calf and mid upper arm circumference | anthropometric measurements | EWGOSP 2 | Handgrip Gait speed | NI | DM 28.6% |
| Karim et al. | 2022 | 32 | EWGOSP 2 | ASMI | Bioimpedance | EWGOSP 2 | Handgrip Gait speed | NI |  |
| Cvjetan et al.* | 2022 | 26 | EWGOSP 2 | ASMI | DXA | EWGOSP 2 | Gait speed | NI |  |
| Karim et al. | 2022 | 37 | EWGOSP 2 | ASMI | Bioimpedance | EWGOSP 2 | Handgrip | NI |  |
| Fonseca et al. | 2022 | 29 | EWGOSP 2 | ASMI | DXA | EWGOSP 2 | Handgrip | NI |  |
| Pinijmung et al | 2022 | 29 | AWGS | SMI | Bioimpedance | AWGS | Handgrip Gait speed | NI |  |
| Fonseca et al. | 2020 | 15 | EWGOSP 2 | ASMI | DXA | EWGOSP 2 | Handgrip | 5 |  |
| Fonseca et al. | 2020 | 66 | Baumgartner | ASM | DXA | EWGOSP 1 | Handgrip | 14 |  |
| Telfer et al. | 2020 | 2 | Baumgartner | ASM | DXA | None |  | NI |  |
| Sun et al. | 2020 | 75 | AWGS | ASMI | DXA | AWGS | Handgrip Gait speed | NI |  |
| Canteri et al. | 2019 | 8 | FHNI | ASM/BMI | DXA | FHNI | Handgrip | NI | HBP 78.5% DM 30.4% |
| Nozaki et al. | 2019 | 20 | AWGS | SMI | Bioimpedance | AWGS | Handgrip Gait speed | NI |  |
| Fonseca et al. | 2019 | 33 | EWGOSP 1 | SMI | DXA | EWGOSP 1 | Handgrip | NI |  |
| Gulyaev et al. | 2019 | 31 | FHNI | ASMI | DXA | None |  | NI |  |
| Emami et al. | 2019 | 30 | Baumgartner | ASMI | DXA | None |  | NI | HBP 59.4% DM 38.2% |
| Dos Santos et al. | 2019 | 75 | Newman | ASMI | DXA | None |  | NI |  |
| Watanabe et al. | 2017 |  | No description | FFMI | Forbes formula | None |  | NI |  |
| Hajahmadi et al. | 2017 | 16 | Baumgartner | ASMI | DXA | None |  | NI |  |
| Molinero-Abad | 2015 | 30 | Baumgartner | ASMI | DXA | None |  | NI |  |
| Obata et al. | 2015 | 17 | EWGOSP 1 | ASMI | DXA | EWGOSP 1 | Handgrip | NI |  |
| Haykowsky et al | 2013 | 25 | Newman | ASMI | DXA | None |  | NI |  |
| Landi et al | 2013 | 5 | EWGOSP 1 | Mid arm circunference | anthropometric measurements | EWGOSP 1 | Handgrip Gait speed | NI |  |

Footnote:

EWGSOP2 = sarcopenia definition proposed by European Working Group on Sarcopenia in Older People in 2018

AWGS = sarcopenia definition proposed by Asian Working Group for Sarcopenia in 2014

Baumgartner = sarcopenia definition proposed by Baumgartner et al. in 1998

EWGSOP1 = = sarcopenia definition proposed by European Working Group on Sarcopenia in Older People in 2010

FHNI = sarcopenia definition proposed by Foundation for the National Institutes of Health in 2014

Newman = sarcopenia definition proposed by Newman et al. in 2003

DXA = dual-energy X-ray absorptiometry

*The Cyvetan et al study was an updated of the Loncar et al study.

4) *Standardize terminology across the manuscript and review grammar.*

We thank the reviewer for the comment. It was done.

5) *The discussion and methods sections could be more concise.*

We shortened the discussion and methods sections.

*Reviewer #3: (No Response)*

*Reviewer #4:*

1) *Could the authors standardize the presentation of the results throughout the manuscript, particularly between the abstract and the results section?*

We thank the reviewer for the comment. We have standardized the data presentation.

2) *Harmonizing the reporting format would enhance clarity and comparability of the findings.*

We thank the reviewer for the comment. We have harmonized the reporting format.

3) *Could the authors provide a more in-depth explanation of the source of heterogeneity observed in the main meta-analysis, especially considering the I² value of 77.2% in the comparison between individuals with heart failure and the control group?*

Unfortunately, due to the small number of studies, we were unable to perform multivariate or subgroup analysis to identify the source of heterogeneity in the meta-analysis comparing people with HF and controls.

*-*

4) *Could the authors elaborate further on the clinical and epidemiological implications of sarcopenia prevalence in individuals with heart failure due to Chagas disease, particularly in light of the scarcity of data in Latin American populations and the known impact of sarcopenia on morbidity and mortality?*

We believe our study is of great importance in identifying the gap in the study of sarcopenia in people with Chagas disease. We understand that our study raises the hypothesis that these patients may be vulnerable to this disorder and that it identifies a potentially important area for research and new investment in Latin America.

5) *Although the leave-one-out analysis was appropriately performed, could the authors offer a clinical interpretation of the influence of studies such as Formiga et al. and Karim et al. on the observed heterogeneity?*

The study by Formiga et al. is a population-based cohort study conducted in Spain, while the study by Karim et al. is a case-control study conducted in a tertiary hospital in Pakistan. We believe that the participant recruitment, location, and the study design may have influenced heterogeneity.

**Summary and General Comments**

*Reviewer #1: (No Response)*

*Reviewer #2:*

1) *This systematic review addresses an important and under-researched topic. The study contributes novel insights into sarcopenia in a neglected population. It highlights a need for tailored clinical approaches in Chagas-endemic regions.
   The authors should be commended for their effort to synthesize the available evidence across multiple studies and apply a structured methodology aligned with PRISMA and GRADE guidelines. The registration of the review protocol on PROSPERO and the inclusion of a meta-regression analysis reflect good scientific rigor. However, the conclusions overreach the presented data, and the methodological heterogeneity significantly limits the strength of the findings. The overstated conclusions are drawn from small and statistically inconclusive subgroups.*

We thank the reviewer for the comment, and we have adjusted our conclusions accordantly.

*Reviewer #3: (No Response)*

*Reviewer #4: (No Response)*

CRUZ-JENTOFT, A. J.; BAHAT, G.; BAUER, J.; BOIRIE, Y. *et al.* Sarcopenia: revised European consensus on definition and diagnosis. **Age Ageing**, 48, n. 1, p. 16-31, Jan 1 2019.

WATANABE, T.; NARUMI, T.; KUBOTA, I. SY4-3 - Impact of Sarcopenia on the Prognosis in Heart Failure. **Journal of Cardiac Failure**, 23, n. 10, Supplement, p. S8, 2017/10/01/ 2017.

ZHANG, Y.; ZHANG, J.; NI, W.; YUAN, X. *et al.* Sarcopenia in heart failure: a systematic review and meta-analysis. **ESC Heart Fail**, 8, n. 2, p. 1007-1017, Apr 2021.
